# Poster Session II - A248 DETAILED COMPARISON OF MUCUS FUNCTION, HOST-MICROBE INTERACTIONS, AND COLITIS SUSCEPTIBILITY IN HUMANIZED MICE LACKING TERMINAL CORE STRUCTURES ON MUCINS

**DOI:** 10.1093/jcag/gwaf042.248

**Published:** 2026-02-13

**Authors:** D Kniffen, M Dirks, E Howard, P Daneshgar, W Zandberg, K Bergstrom

**Affiliations:** Biology, The University of British Columbia Okanagan, Kelowna, BC, Canada; Biology, The University of British Columbia Okanagan, Kelowna, BC, Canada; Biology, The University of British Columbia Okanagan, Kelowna, BC, Canada; Biology, The University of British Columbia Okanagan, Kelowna, BC, Canada; Biology, The University of British Columbia Okanagan, Kelowna, BC, Canada; Biology, The University of British Columbia Okanagan, Kelowna, BC, Canada

## Abstract

**Background:**

Mucus maintains colonic homeostasis by forming a barrier around the fecal mass to limit microbial contact. Mucus, a polymer of MUC2, is ∼80% carbohydrate due to dense O-glycans composed of Gal, Fuc, GalNAc, GlcNAc and Sialic acid (Sia), added by glycosyltransferases in the Golgi. Each O-glycan derives from a core (e.g. core 1 Galβ1,3GalNAcαSer/Thr) that can be branched and capped by Fuc or Sia. Mutations in enzymes controlling fucosylation, sialylation, and core synthesis are linked to IBD; however, their relative contribution to mucus function in a humanized setting remains unclear.

**Aims:**

To assess how lthe oss of fucosylation, sialylation, or core glycan synthesis impacts mucus structure and colitis susceptibility in mice with humanized microbiota and diet.

**Methods:**

Fut2-/- (lacking α1,2Fuc), Tamoxifen-inducible (TM)-IEC Slc35a1-/- (lacking the activated nucleotide Sia transporter needed for sialylation), and TM-IEC DKO (lacking core 1 and 3 synthases and thus all major complex O-glycans) mice were colonized with human microbiota and maintained on a human-profile diet, with TM used to induce epithelial deletion where applicable. 3 weeks later, mucus structure, microbiota (16S rRNA profiling), histology, and gene expression were analyzed in proximal/distal mucosal and luminal compartments. Mucus glycan profiles were determined by glycomics.

**Results:**

Lectin staining with *Ulex europaeus* agglutinin 1 (UEA1, targeting Fucα1,2Gal) *Sambucus nigris* agglutinin 1 (SNA1, targeting Siaα2,6Gal) and confirmed selective loss of α1,2Fuc (UEA1-) in humanized (Hu):Fut2-/- mice with intact Sia (SNA+), and loss of Sia (SNA-) but not α1,2Fuc in Hu:TM-IEC Slc35a1-/- mice. Glycomics confirmed absence of fucosylated or sialylated structures respectively. Fut2 loss led to an altered niche layer but preserved barrier integrity and did not induce colitis. In contrast, loss of Sia expanded the niche layer, reduced the barrier, and allowed microbial penetration. TM-DKO mice showed complete mucus degradation despite goblet cells retaining N-glycan Fuc and Sia. All mutants displayed microbiota compositional shifts. Histologically, Hu:Fut2-/- remained normal, while Hu:TM-IEC Slc35a1-/- developed spontaneous colitis. Disease was most severe in Hu:TM-DKO mice, correlating with mucus loss.

**Conclusions:**

In a human-relevant ecosystem, terminal sialylation may be more critical than fucosylation for maintaining mucus integrity and preventing colitis but full homeostatic function requires coordinated contributions of core and capping glycan structures.

**Funding Agencies:**

Weston Family Foundation

